# Dietary encapsulated essential oil mixture influence on apparent nutrient digestibility, serum metabolic profile, lymphocyte histochemistry and intestinal morphology of laying hens

**DOI:** 10.5713/ab.21.0275

**Published:** 2021-09-15

**Authors:** Cavit Arslan, Abdurrahman Pirinç, Nizamettin Eker, Emrah Sur, İlknur Ündağ, Tansu Kuşat

**Affiliations:** 1 Department of Animal Nutrition and Nutritional Disease, Faculty of Veterinary Medicine, Selcuk University, 42100 Campus, Selcuklu, Konya, Turkey; 2 Healty Science Institute, Selcuk University, 42100 Campus, Selcuklu, Konya, Turkey; 3 Department of Histology and Embryology, Faculty of Veterinary Medicine, Selcuk University, 42100 Campus, Selcuklu, Konya, Turkey

**Keywords:** ACP-ase, Alpha-naphthyl Acetate Esterase (ANAE), Blood, Essential Oils Mixture, Intestinal Morphology, Laying Hens, Nutrient Digestibility

## Abstract

**Objective:**

The study aimed to evaluate the effects of a mixture of encapsulated essential oils (EOs) addition on nutrient digestion, serum biochemical parameters, peripheral blood alpha-naphthyl acetate esterase (ANAE), and acid phosphatase (ACP-ase) positive lymphocyte ratios and intestinal morphology in laying hens.

**Methods:**

A total of 320 laying hens of 48-wk-old were randomly allotted into 4 treatment groups with 10 replicates of 8 birds in each replicate. The birds were fed a basal diet (control) or the diet added with mixture of EOs (which consist of eugenol, nerolidol, piperine, thymol, linalool, and geraniol) at 50, 100, and 200 mg/kg for period of 84 days.

**Results:**

The addition of EOs at 100 or 200 mg/kg increased the dry matter, organic matter, and crude protein digestion as compared to control. The addition of all doses of EOs did not affect serum gamma glutamyl transferase, alanine aminotransferase, and P but increased serum asparate aminotransferase (AST) concentration. The addition of 200 mg/kg EOs increased serum creatinine, while 100 mg/kg decreased Ca concentration. The addition of 100 and 200 mg/kg EOs generally improved ANAE and ACP-ase positive peripheral blood lymphocyte ratios and intestinal morphology.

**Conclusion:**

It can be concluded that, the addition of 100 or 200 mg/kg encapsulated EOs generally increased apparent nutrient digestion and serum AST concentration, improved ANAE and ACP-ase positive peripheral blood lymphocytes and intestinal morphology in laying hens.

## INTRODUCTION

Various subtherapeutic antibiotics have been used as feed additives since 1940 to enhance growth performance and prevent livestock disease, particularly in poultry. The uses of antibiotics in feed have been questioned due to the potential development of resistance in pathogenic bacteria and antibiotic residues in the animal tissues. Due to concern of transmission of this resistance to human pathogens through the food chain, all antibacterial feed additives are banned in the European Union in 2006. After the ban of antibiotics as feed additives, research has intensified on other feed additives of natural and herbal origin that do not leave residues in animal tissues. One of those alternatives is essential oils (EOs). Essential oils are volatile secondary metabolites abundant in aromatic plant families such as *Lamiaceae* and *Apiaceae*, containing many compounds such as monoterpenes and sesquiterpenes [1]. Aromatic plants contain a variety of functional bioactive compounds. Major compounds present in EOs are linalool (67.70%), a-pinene (10.5%), g-terpinene (9.0%), geranyl acetate (4.0%), camphor (3.0%), and geraniol (1.9%) [2]. It is recommended to use EOs in an encapsulated form because EOs are volatile compounds, and they are also sensitive to heat, light, UV light, moisture, pH, metals and atmospheric oxygen, and deteriorate quickly leading to a decrease in active ingredients depending on the storage time [3]. Additionally, encapsulated EOs may slowly release their contents at controlled rates under specific conditions [4], which may prove more effective than their non-encaptulated form. Furthermore, it was suggested that the type of EOs has major influence on the growth performance of broilers, which reflects that different EOs are not effective equally for different response variables [5] so mixtures of EOs may be more effective as compared to the individual EO.

The mode of action of the bioactive compounds in EOs has not been fully explained. Various mechanisms have been suggested for a mode of action of EOs on nutrients digestion. Several authors have indicated that the use of EOs stimulates digestive enzyme (such as trypsin and amylase) activity [6], others a production and induction of a higher secretion of bile acids [7]. It has been reported that the addition of the various amount of EOs to the laying hen’s diet did not affect dry matter (DM) digestion [8,9]. Radwan Nadia et al [10] detected that the addition of 0.5% to 1.0% of EOs from thyme, oregano, rosemary, or curcuma to laying hen’s diet did not affect the digestibility of organic matter (OM). Yu et al [9] stated that adding different doses of anise oil (200, 400, and 600 mg/kg) increased the digestibility of OM in laying hens. Although it has been determined in some studies that the addition of EOs increased digestibility of crude protein (CP) in laying hens [8,9], whereas in another research no effect was found [10].

It was stated that the addition of thymol [11] and oregano EOs [12] to the laying hens’ diets did not affect serum Ca and P concentrations.

Essential oils also have anti-inflammatory and immune-modulating properties, it was shown that clove oil, a major component of eugenol, increased humoral responses in human cell cultures [13]. Clove is also known as an immunomodulator that can induce lymphocyte proliferation and macrophage production [14]. Alpha-naphthyl acetate esterase (ANAE) demonstration has been widely used to differentiate T lymphocytes from B lymphocytes in some animal species, including chickens [15]. Positivity of acid phosphatase (ACP-ase) enzyme may also be used to identicate B lymphocytes in chicken [16].

It was demonstrated that the addition of oregano EOs to laying hens diets improved intestinal morphology [17], different levels of anise and thyme addition to broilers diet also induced an increase in duodenum, jejunum and ileum wall thickness [18].

The effect of EOs on apparent nutrient digestibility, blood serum metabolic profile and small intestinal morphology has been investigated in some previous studies. There is no complete accordance between the results obtained from these studies. The purpose of this study was to examine the effects of encapsulated EOs mixtures on the apparent digestibility (AD) of nutrients, blood serum metabolic profile, peripheral blood lymphocyte (PBL) histochemistry and gut morphology of the small intestine in laying hens.

## MATERIALS AND METHODS

This study was carried out at Prof. Dr. Humeyra Ozgen Research and Application Farm, Faculty of Veterinary Medicine, Selcuk University (Konya, Turkey), in accordance with guidelines of the Selcuk University Animal Experiments Production and Research Committee (Date 27.03.2020, No: 2020/36).

### Experimental birds, diets, and management

A total of 320 Nick Brown laying hens 48 weeks’ old were used in this study. They were randomly divided into 4 treatment groups, each containing 80 birds. Ten replicates were set for each treatment, with 8 birds in each replicate.

The basal diet was formulated to meet nutrient requirements of Nick Brown laying hens recommended by NRC [19], and its ingredients and composition are shown in Table 1. A basal diet was supplemented with a commercial encapsulated EOs mixture (Agoline Poultry: Agolin S.A., Rte de la Picarde 20, 1145 Bière, Switzerland) at the concentrations of 0 (Control), 50 (EO-50), 100 (EO-100), and 200 mg/kg (EO-200) respectively. The mentioned doses were based on the product. According to the declaration of the manufacturer, it is coated with hydrogenated sunflower oil by the spray cooling technique. The main ingredients of Agoline Poultry are as follows; 50.00% sunflower oil as carrier, approximately 20.00% active compounds consisting of linalool, eugenol, thymol, geraniol, nerolidol and piperine, and 30.00% other compounds such as organic acids, silica and syntetic antioxidants.

All hens were kept individually (one bird per cage) in environmentally controlled caged houses (30× 45×50 cm) with temperature maintained at approximately 20°C to 24°C. The house had controlled ventilation and lighting (16 L:8 D). All hens were fed with diets in granular form and water for *ad libitum* during the study. The study lasted for 12 weeks (48 to 60 weeks).

### Nutrient digestibility

For the measurement of the nutrients’ AD, 2 birds from each replicate (totally 20 birds per treatment group, 80 birds in total) were randomly selected on the 28th, 56th, and 84th day of the study. Excreta samples of each bird were collected and immediately stored at −20°C. During collection, care was taken to avoid contamination from feathers, feed, and foreign materials. Excreta were dried at 80°C until constant weight. Diet and excreta were ground to pass through a 1 mm screen. Samples of the feed and excreta were analyzed for DM, CP (CP: N×6.25), ash and acid insoluble ash, as described by the AOAC [20]. The AD of DM, OM, and CP was determined by the acid insoluble ash ratio and AD of the nutrients was calculated using the following equation [21]:


AD=100-[(feed indicator %/excreta indicator %)×(excreta nutrient %/feed nutrient %)×100]

### Blood sampling and laboratory analysis

On the 84th day of the study, ten birds from each group (1 bird from each replicate) were randomly selected for blood collection, and blood was drawn from wing vein (*Vena subcutenea ulnaris*) using sterilized needles and syringes in vacutainer tubes for serum collection. Blood samples were allowed to standard for 2 h at room temperature to enable proper clotting. The samples were then centrifuged at 3,000 g×for 10 minutes. Serum gamma glutamyl transferase (GGT), asparate aminotransferase (AST), alanine aminotransferase (ALT), creatinine, Ca, and P concentration was measured using commercially available test kits at an autoanalyzer (GEM Primer Plus 3000, 74351, Blood gas/Electrolyte Analiser, Model 5700; Instrumental Laboratories, Bedford, MA, USA).

### Histochemistry

#### Blood sampling

On the 84th day of the study, one bird from six subgroups (1, 2, 3, 4, 5, and 6) was selected randomly on the basis of average live body weight of each subgroup for peripheral blood samples (6 birds from each group, totally 24 birds). The birds sacrificed by cervical dislocation and peripheral blood samples were collected during the cervical dislocation. Air-dried blood smears were used for ANAE and ACP-ase histochemistry and fixed in cold glutaraldehyde-acetone solution at −10°C for 3 min rinsed three times in distilled water.

#### Demonstration and evaluation of histochemistry

Histochemical demonstrations of ANAE and ACP-ase were performed according to Maiti et al [15] and Sur and Celik [16], respectively. In the blood smears examined, some of the PBLs that were ANAE-positive showed one to three dot-like brown-reddish granules. Most of the PBL had strong ACP-ase positivity. The PBL containing one to three red-pinkish cytoplasmic granules was considered ACP-ase positive. In smears stained for ANAE and ACP-ase, 200 lymphocytes were counted, and positive staining rates were calculated as the percentage of counted cells.

### Intestinal morphology

Same 24 birds, which were used for peripheral blood sampling, were also used for intestinal histology. For this aim, after killing the birds whole gastrointestinal tracts were immediately removed and then 2 cm of intestinal segments were cut from the duodenum, jejunum and ileum. The segment of the duodenum was collected from 3 cm from the gizzard-duodenal junction point, whereas the jejunum segment was removed just above the Meckel diverticulum. Ileum samples were taken above the ileo-cecal junction [22]. All tissue samples were washed with pre-cooling physiological saline, and their contents were emptied without damaging their mucosa.

Tissue samples were fixed in 10% buffered formaldehyde for 1 week. After the routine histological process, tissue samples were embedded in paraffin by using standard methods. From all paraffin blocks, 3 serial sections of 6 μm thickness were taken. The sections were stained with Crossmon’s triple stain [23] to determine the general histological structure and carried out the histometric measurements while periodic acid Schiff reaction [24] was performed to count goblet cell.

Measurements were made on randomly selected well-orientated 5 villi from different parts of 3 serial sections of each bird. Villus heights (VH) were measured as the length from the tip to the base of villi. At the same time, the crypt depths (CD) were evaluated as the distance between the villi from the invagination started to the bottom end of the Lieberkühn glands. Villus widths (VW) were obtained by measuring at the half height point of the villi, while the thickness of the tunica muscularis (TTM) was determined by measuring the circular and longitudinal muscle layers together. The goblet cell counts (GCC) were obtained in 10 different areas of 100 μm line on sections performed PAS reaction.

All specimens were examined under the light microscope (Leica DM2500; Leica Microsystems GmbH, Wetzlar, Germany) and were photographed by a digital camera (Leica DFC 320) and recorded digitally for histometric analyses.

### Statistics

All data were analysed by one-way analysis of variance (ANOVA) using SPSS version 16.0 statistic software (SPSS Institute Inc., Chicago, IL, USA). The significance of the differences among the groups was assessed by Duncan’s multiple range test. Orthogonal polynomial contrasts were used to asses the significance of linear and quadratic models to describe the response of the dependent variable to a rising EOs levels. The difference between the nutrient digestibilities of the same group on the 28th, 56th, and 84nd days of the study was tested with the general linear model-repeated measures. The criterion of statistically significant for all data was at p values less than p<0.05.

## RESULTS

### Apparent nutrient digestibility

The effect of dietary EOs on apparent nutrient digestibility is shown in Table 2. The AD of DM in the EO-200 group was higher than the other groups on the 28th day of the study (ANOVA, p<0.001; linear, p<0.001; quadratic, p<0.01), it was higher in the EO-100 and EO-200 groups than the Control group on the 56th and 84th day of the study (ANOVA, p<0.001; linear, p<0.001). When the AD of DM on the 28th, 56th, and 84th days of each group is compared within itself, it was higher in the EO-50 and EO-100 groups on the 56th day of the study than on the 28th and 84th d (p<0.01). Apparent digestibility of DM in the EO-200 group on the 84th days of the study was significantly higher than on the 28th and 56th day of the study (p<0.01).

The AD of OM in the EO-100 and the EO-200 groups were higher than the Control group on the 28th, 56th, and 84th days of the study (ANOVA, p<0.001; linear, p<0.001). When the apparent OM digestibility on the 28th, 56th, and 84th days of each group is compared within itself, it was higher only in the EO-50 group on the 56th day of the study than on the 28th and 84th d (p<0.01).

The AD of CP in the EO-100 and EO-200 groups was higher than the Control group on the 28th, 56th, and 84th days of the study (ANOVA, p<0.001; linear, p<0.001). When the apparent CP digestibility on the 28th, 56th, and 84th days of each group was compared within itself, it was higher on the 56th day of the study in the EO-50 group than the 28th and 84th d (p<0.05). Apparent digestibility of CP in the EO-100 group on the 56th day of the study was higher than on the 28th d (p<0.05), it was higher in the EO-200 group on the 84th day of the study than on the 28th d (p<0.001).

### Blood serum parameters

The effect of dietary EOs on serum parameters is presented in Table 3. There were no statistical differences in the concentrations of GGT, ALT, and P among the groups. The concentration of AST was higher in all EOs groups than in the control (ANOVA, p<0.05; linear, p<0.05). The concentration of creatinine in the EO-200 group and Ca in the EO-50 group were higher than the other groups (for creatinine ANOVA, p<0.001; linear, p<0.001; for Ca ANOVA, p<0.01; linear, p<0.001; quadratic, p<0.001).

### Histochemical findings

The mean ratios of the ANAE and ACP-ase positive PBL are shown in Table 4. The ANAE positive lymphocyte ratio was higher in all EOs groups than the control (ANOVA, p<0.001; linear, p<0.001; quadratic, p<0.01). Although there were no statistical differences among ACP-ase ratios of the groups, it was numerically higher in the EO-100 group than the other groups (ANOVA, p>0.065).

### Intestinal morphology

The effects of EOs on histometric parameters in the small intestine segments (i.e., VH, VW, CD, TTM, and the GCC in duodenum, jejunum, and ileum) of laying hens are presented in Table 4. The VH of duodenum in the EO-100 and EO-200 groups was higher than those of other groups (ANOVA, p<0.001; linear, p<0.001). The VW of duodenum was lower in the EO-50 and EO-100 groups than in the other groups (ANOVA, p<0.001; quadratic, p<0.001). The CD of duodenum were higher in the EO-100 and EO-200 groups than the other group (ANOVA, p<0.001; linear, p<0.001). The TTM of duodenum was lower in the EO-50 and EO-200 group than in the other groups (ANOVA, p<0.001). The GCC of duodenum was lower in the EO-200 groups as compared to other groups (ANOVA, p<0.001; linear, p<0.001; quadratic, p<0.001).

The VH of jejunum was lower in the EO-50 and EO-200 groups than the other groups (ANOVA, p<0.001; linear, p<0.05). The VW of jejunum was wider in the EO-100 than the Control and EO-50 groups (ANOVA, p<0.05; linear, p<0.01). The CD of jejunum in the EO-100 group was higher than the other groups (ANOVA, p<0.001; linear, p<0.05; quadratic, p<0.001). The TTM of jejunum was lower in the EO-200 group than the other groups (ANOVA, p<0.001; linear, p<0.001), while the GCC was higher in all EO groups compared to control (ANOVA, p<0.001; linear, p<0.001; quadratic, p<0.001).

The addition of EOs did not affect VH of ileum, while VW increased in all EO groups compared to control (ANOVA, p<0.001; linear, p<0.001; quadratic, p<0.001). The CD of ileum was higher in the EO-200 group than the EO-50 and Control groups (ANOVA, p<0.01; linear, p<0.001). The TTM of ileum was lower in the EO-100 group than those of other groups (ANOVA, p<0.001; quadratic, p<0.001). The GCC of ileum was quadratically higher in the EO-100 group than the Control (quadratic, p<0.05).

## DISCUSSION

### Apparent nutrient digestibility

Apparent digestibility of DM varied between 69.47% and 75.97% in all groups on the 28th, 56th, and 84th days of the study (Table 2), which remained within the previously reported ranges [9,25,26]. The addition of 200 mg/kg EOs to diets on the 28th day of the study or 100 and 200 mg/kg on the 56th and 84th day of the study significantly increased DM digestion compared to Control. The increased DM digestion in the EO-100 and EO-200 groups indicated that the addition of high amounts of EOs (100 and 200 mg/kg) increased DM digestion, whereas low amount (50 mg/kg) remain unchanged. In a previous study in broilers, addition of 200 mg/kg EOs or 5,000 mg/kg of Labiatae extract determined that Labiatae extract increased DM digestion during starter period, while both Labiatae extract and EOs increased it during finisher period [25]. It was also determined that the addition of 200, 400, and 600 mg/kg anise oil [9] and 0.5% and 1.0% of different EOs (i.e., thyme, oregano, rosemary, and curcuma) did not affect DM digestion in laying hens [10]. When the AD of DM at 28th, 56th, and 84th days of each treatment is evaluated within itself, there was an increase in the EO-50 and EO-100 groups on the 56th day of study, and also in the EO-200 group at the 84th day of study (Table 2).

In the present study, AD of OM ranged from 73.54% to 79.99% in all the groups on 28th, 56th, and 84 th days of the study (Table 2), which are consistent with other studies results [9,26]. The increased OM digestion in the EO-100 and EO-200 groups on the 28th, 56th, and 84th days of the study (Table 2) as compared to Control indicated that 100 or 200 mg/kg EOs addition positively affected OM digestion in laying hens. Similarly, it was reported that the addition of 400 and 600 mg/kg EOs increased OM digestion compared to control in laying hens [9]. It was also detected that the addition of a blend of EOs at 80, 125, and 250 mg/kg had significant quadratic effects on OM of diets’ total tract metabolic efficiency in male broilers [26]. Contrarily, it has been reported that the addition of 0.5 and 1.0 % of various EOs to laying hen diets did not affect OM digestion [10]. When the AD of OM at 28th, 56th, and 84th days of each treatment is evaluated within itself, there was an increase in OM digestion only in the EO-50 group on 56th day of study (Table 2).

In this study, the AD of CP varied between 50.08% and 57.69% in all groups on the 28th, 56th, and 84th days of the study (Table 2). These results are in accordance with some previous studies [8,25] but lower than those of others [9,10, 26,27]. In general, it was noted that the addition of EOs to the diets increased the CP digestion by increasing the dose, in other word EOs addition showed a linear affect on the CP digestibility. This increase was significantly higher in the 100 and 200 mg/kg EOs added groups than the Control. The results indicated that a higher dose of EOs (100 and 200 mg/kg) positively affected CP digestion in laying hens. Similarly, it was reported that the addition of 100 mg/kg [8] or 400 mg/kg [9] EOs to the laying hen diets increased CP digestion, while it did not change by lower or upper doses than above mentioned. Contrarily, it has been reported that the addition of 0.5% and 1.0% of EOs did not affect CP digestion in laying hens [10]. When the CP digestibility at 28th, 56th, and 84th days of each treatment is evaluated within itself, it reflects that in general CP digestion reached the highest ratio on 56th day of study (Table 2).

Based on the apparent nutrient digestibility data presented in this study, a generally improved nutrient digestibility could be postulated for the 100 and 200 mg/kg EOs treatments. Possible reasons for the increase in apparent nutrient utilization of 100 and 200 mg/kg EOs added groups might be associated with better secretion of digestive enzymes. One of the main active compound of EOs used in this study is piperine, which stimulates digestive enzymes [7,28]. Gao et al [29] found that encapsulated blends of EOs (150, 200, and 250 mg/kg diet) enhanced jejunal mucosal α-amylase and chymotrypsin activity, improved intestinal morphology, and promoted the performance of broilers. A study conducted by adding 50, 100, and 150 mg/kg oregano EOs to laying hen diets determined that 100 mg/kg increased amylase and trypsin activity but did not affect lipase activity. It was also observed that EOs had positive effects on increasing the secretions of digestive enzymes and intestinal mucous in the gut, thus improving the digestibility of the feeds [7,30]. Therefore, the improvement of nutrient utilization in this study may be caused by the stimulative effect of the EOs on the secretions of the endogenous digestive enzymes and the increased absorption surface area of the small intestine (Table 4).

### Blood serum parameters

The biochemistry evaluation of liver enzymes (such as AST, ALT, GGT) gives evidence about metabolic disorders caused by diseases or nutritional deficiency that influence hepatic activity. The GGT is an enzyme found in many organs throughout the body, with the highest concentrations found in the liver. It is elevated in the blood in most diseases that cause damage to the liver or bile ducts. The GGT can indicate cholestasis and biliary duct proliferation in the chicken liver [31]. In this study, serum GGT concentration varied between 26.1 and 26.9 U/L in all groups (Table 3). Our results are similar to results of Gonçalves et al [31] who reported 17.37 to 35.02 U/L before and during the peak of egg production respectively.

The AST enzyme is considered a responsive marker in liver disorder in chicken, even if it is a nonspecific parameter [31]. In the present study, serum AST concentration ranged between 147 and 167 U/L in all groups (Table 3), which is similar to the results of Lokaewmanee et al [32], higher than Mousavi et al [33] and Abo Ghanima et al [34] and lower than that of Bolukbası et al [35]. Our results indicated that the addition of all EOs doses increased the serum AST concentration as compared to Control. The increase in AST concentration in all the EOs added group might be due to increased feed intake because of high egg production during the study period. Gonçalves et al [31] reported that increasing feed intake causes a higher overload in the liver of commercial laying hens; thus, serum levels of AST enzyme increase. Enhanced AST and ALT activities are generally considered a biomarker of hepatic damage or dysfunction [36], tissue disturbance, and increased membrane permeability and liver enzymes escaping to blood serum [31]. In this study, no interpretation could be made because a histo-pathological examination was not performed in the liver tissue. Contrarily, in many previous studies, it has been reported that the addition of EOs to diet did not affect [10,17,32,35] or decreased serum AST concentration in laying hens [33,34].

In this study, serum ALT concentration changed between 1.10 and 1.50 U/L among the groups (Table 3), which are similar to results of previous studies [32,35]. Our results indicated that the addition of all doses of EOs to the diet did not affect the serum ALT concentration. Similarly, in previous studies conducted in laying hens, it was determined that the addition of EOs did not affect the serum ALT concentration [10,17,32]. Contrarily, it was found that 100 and 200 mg/kg EOs mixture [33] and 300 mg/kg rosemary or cinnamon decreased serum ALT concentrations in laying hens [34].

Creatinine, a by-product of phosphocreatine breakdown in skeletal muscle, is an important indicator of protein metabolism [37] and an index of renal function [38]. Its blood levels are related to muscle mass, age, physical activity, and diet [39]. In the current study, serum creatinine concentration ranged between 0.39 and 0.45 mg/dL among the groups (Table 3), which is similar to the results of other studies [34, 38]. Our results indicated that only 200 mg/kg EOs addition to the diet increased serum creatinine concentration when compared to other groups. The higher serum creatinine concentration in the EO-200 group might be related to the higher apparent CP digestion in this group (Table 2). In many previous studies, it was determined that the addition of EOs did not affect the serum creatinine concentration in laying hens [17,32,34].

In this study, serum Ca concentration varied 29.2 to 33.3 mg/dL all the groups (Table 3), which is similar to the results of previous studies [17,32] but higher than that of Bolukbası et al [35]. Our results indicated that the addition of 50 mg/kg EOs to the diet increased serum Ca concentration, 100 mg/kg decreased and 200 mg/kg unchanged as compared to Control. The result reflected that 50 mg/kg EOs improved the bio-availability of Ca in laying hens. Similarly, it was determined that 400 and 600 mg/kg oregano EOs [17] and 300 mg/kg rosemary or cinnamon [34] increased serum Ca concentration, whereas 0.5 and 1.0 mg/kg bergamot oil decreased it [35], but 250 mg/kg thymol [11] and 24 mg/kg oregano EOs [12] did not change serum Ca concentration in laying hens. It was reported that EOs could positively affect serum Ca and P concentration probably by improving intestinal morphology and oxidative stress or by increasing intestinal pH [40]. However, Yoon et al [41] indicated that piperine, which is one of the active compounds of used EOs in this study, inhibits the activity of Ca2-ATP-ase enzyme in transporting the calcium ions across the cell membrane that will decrease calcium.

In the present study, serum P concentration ranged between 5.31 to 5.99 mg/dL among the groups (Table 3), which is similar to the results of previous studies [17,35] but higher than those of others [11,12,32,34]. Our results indicated that the addition of 50, 100, and 200 mg/kg EOs to the diet did not affect serum P concentration. Similarly, it was reported that different EOs supplementation to diet did not influence serum P concentration in laying hens [11,12,32,35]. Contrarily, it was determined that EOs addition increased serum P concentration in laying hens [17,34].

In general, possible reasons for some of the differences in blood serum parameters between this study and other studies might be due to the dose of EOs used in diets, the different active substances in EOs, the strain and age of the birds used in the research, the nutritional contents of the diets and the differences in feeding programs.

### Histochemistry

Lymphocytes have a vital role in body protection against infection and are known as mediator cells of the adaptive immune response and are divided into B- and T-lymphocytes. B-lymphocytes are responsible for antibody-production, and their maturation process occurs in the bone marrow and bursa of Fabricius in mammals and avian species, respectively. However, T-lymphocytes are responsible for cellular immunity, and the maturation of T lymphocytes occurs in the thymus in both mammals and avian species [42]. Awaad et al [43] demonstrated that some EOs stimulated the immune response in chickens. In this study, it was observed that 50, 100, and 200 mg/kg EOs addition increased the ANAE positive lymphocyte ratios (Table 4). As known ANAE positive lymphocyte is accepted T-cell the increase may reflect an improvement of cellular immunity. We could not compare our ANAE positivity results due to the lack of study. However, Lee et al [44] observed proliferation of spleen lymphocytes and increasing peripheral blood T-lymphocytes in chicken fed a diet containing phytogenic mixture.

The ACP-ase positive lymphocyte is accepted B cell in chicken, which is responsible for humoral immunity [16]. Our result showed that although there was no statistical difference, 100 and 200 mg/kg EOs addition numerically increased the ratio of ACP-ase positive lymphocyte (p<0.065). Lee et al [44] determined that there were higher serum antibody titers against some certain *Eimeria* species in chicken fed a diet containing phytogenic additives. Although the immunomodulatory effects of EOs are known, the mechanism of their biological action still remains obscure. Wael et al [14] have reported that *Syzygium aromaticum* leaf extract increased the proliferative activity of lymphocytes in Balb/c mice infected with *Salmonella typhimurium*. Kim et al [45] claimed that the addition of some phytogenic additives altered gene expression of certain cytokines in the intestinal intraepithelial lymphocytes of chickens.

### Intestinal morphology

Generally, it is accepted that increased thickness of the intestinal mucosa, especially VH and VW, are correlated by an increased digestive and absorptive function of the small intestine due to enlarged absorptive surface area. Our results indicated that the addition of 100 and 200 mg/kg EOs increased the VH of the duodenum, while 50 and 200 mg/kg decreased the VH in the jejunum, but none of the EOs affected VH in the ileum (Table 4). The addition of 50 and 100 mg/kg EOs decreased VW in the duodenum; only 100 mg/kg EOs increased VW of jejunum. The VW of ileum was higher in the all EOs group than the Control. Our results on the VH and VW are in accordance with Gul et al [17] who used oregano oil in laying hens. Similarly, Sur et al [22] demonstrated that especially high level mint addition increased VH but decreased VW in all small intestinal segments in Japanese quails.

Intestinal glands, also called Lieberkühn crypts, extend down from the base of the villi to the muscular mucosa. Undifferentiated epithelial cells located in these glands enlarge intestinal mucosal surface area and renew intestinal epithelium by dividing and migrating to villi [18]. Many authors have shown that an increase in the depth of the crypts, which serve as a cell source for the intestinal epithelium, is an indicator of cell renewal rate. There are opposing views about CD, and some researchers claimed that the deep crypts also reflect the long villi [46,47] while some others argued that increasing CD shows faster cellular turnover in response to inflammation or epithelial cell shedding [29]. In general, the addition of 50 mg/kg EOs causes a decrease in CD in all segments of the small intestine, whereas 100 mg/kg increased. However, 200 mg/kg EOs increased CD of duodenum and ileum but decreased of jejunum. In agreement with this study, it was reported that the addition of organic acids and EOs improved the villi number, VH and CD of the small intestine in broiler chicken [48]. It was also demonstrated that a high level mint addition increased CD in all segments of the small intestine in Japanese quails [22]. However, in another study it was determined that EOs addition did not influence VH and CD, but VH:CD ratio was higher in broiler chickens [49]. Gao et al [29] found that the mixture of EOs and organic acids decreased CD-length in the duodenum and jejunum but increased VH:CD ratio of 21-day-old broilers as compared to control. In an experimental study it was found that the addition of EOs mixture increased VH and VH:CD ratios of jejunum [50].

Tunica muscularis is responsible for intestinal motility resulting in peristalsis, which is an involuntary movement. Peristalsis has a crucial role in the passage of food mixture along the intestinal tract. This movement increases the enzymatic activity on nutrients by mixing of contents; finally these processes improve the absorption of nutrients [51]. In the present study, the tendency of a decrease in TTM of all small intestinal segments was generally observed. Contrarily, in many studies adding high level oregano oil or mint addition increased the TM layer [17,22].

Goblet cells are scattered within the enterocytes, producing the mucin [18]. Mucin coats the gastrointestinal tract, and it is accepted as the first-line host defense. These cells in the intestinal mucosa provide lubricity via mucin which also acts as a protective barrier against pathogenic microorganisms [52]. Gao et al [46] reported that the increase in the GCC may be related to the increase in the CD, which reflects epithelial regeneration. Additionally, Wang et al [53] reported that an increase in the GCC due to *Bifidobacteria* can cause the secretion of mucin-2 and may enhance the intestinal mucosal immune function of birds. However, it was suggested that GCC increase is related to mucosal damage. In addition, the increase caused decrease nutrient absorption and increased extra energy consumption [54]. In this study, there was a decrease in GCC of the duodenum in the EO-200 group as compared to other groups (Table 4). However, GCC in the jejunum in all EOs groups was higher than control. Generally, GCC increased tendency depending on the EOs level in the ileum, but it was only higher in the EO-100 group than the control. Similarly, Hamedi et al [47] has shown a significant increase in GCC of jejunum and ileum of birds fed with diets containing sunflower meal. Sur et al [22] also demonstrated that high level mint supplementation increased GCC in the ileum of Japanese quails.

## CONCLUSION

In conclusion, the addition of 100, and especially 200 mg/kg, of a mixture of encapsulated EOs in laying hens diet, improved DM, OM, and CP digestion, but 50 mg/kg did not influence these parameters. The addition of 50, 100, and 200 mg/kg EOs did not affect serum GGT, ALT, and P concentration but increased the serum AST concentration. The addition of 50 mg/kg and 200 mg/kg EOs increased Ca and creatinine concentration, respectively. But 100 mg/kg decreased serum Ca concentration. The addition of EOs to laying hens diet generally increased the ANAE and ACP-ase positive PBL ratio, and this increase might be an immunomodulatory effect in laying hens. There was great variability in small intestinal morphology depending on the EOs treatment. When the intestinal histology findings are evaluated as a whole, it can be said that the addition of 100 and 200 mg/kg EOs improved the intestinal morphology, and this improvement could positively affect nutrient absorption. It can be concluded that, the improved nutrient digestion, ANAE and ACP-ase positive lymphocyte ratio, intestinal morphology addition of a mixture of encapsulated EOs 100 and 200 mg/kg (which containing eugenol, nerolidol, piperine, thymol, linalool, and geraniol as active compounds) has a beneficial effect in laying hens. Futher studies should be done to find the most optimum dose rate of encapsulated EOs between 100 and 200 mg/kg.

## Figures and Tables

**Table 1:** Ingredients and nutrient composition of the basal diet (%)

Item	Dietary EOs concentration (mg/kg)^1)^			

	0	50	100	200
Ingredients				
Corn	50.00	50.00	50.00	50.00
Barley	7.15	7.15	7.15	7.15
Wheat	5.20	5.20	5.20	5.20
Vegetable oil	2.50	2.495	2.49	2.48
Soybean meal (44% CP)	20.00	20.00	20.00	20.00
Sunflower meal	3.77	3.77	3.77	3.77
Limestone	8.71	8.71	8.71	8.71
Dicalcium phosphate	1.80	1.80	1.80	1.80
Salt	0.34	0.34	0.34	0.34
DL-Methionine	0.09	0.09	0.09	0.09
L-Threonine	0.04	0.04	0.04	0.04
Premix^2)^	0.40	0.40	0.40	0.40
EOs mixture	-	0.005	0.01	0.02
Chemical composition, in DM				
DM	91.50	91.50	91.50	91.50
ME (MJ/kg)^3)^	11.51	11.51	11.51	11.51
Organic matter	89.44	89.44	89.44	89.44
Crude protein	18.71	18.71	18.71	18.71
Calcium^3)^	3.83	3.83	3.83	3.83
Available phosphorus^3)^	0.41	0.41	0.41	0.41
Methionine+cystine^3)^	0.62	0.62	0.62	0.62
Lysine^3)^	0.75	0.75	0.75	0.75
Threonine^3)^	0.61	0.61	0.61	0.61
Tryptophan^3)^	0.20	0.20	0.20	0.20

EOs, essential oils; CP, crude protein; DM, dry matter; ME, metabolizable energy.

1) Control group was fed the basal diet. Treatment groups were fed same basal additonaly the diet supplemented with 50, 100, and 200 mg/kg of EOs, respectively.

2) Supplied per kg of diet: Vitamin A, 12,200 IU; cholecalciferol, 4,200 IU; vitamin E, 30 IU; vitamin K3; 4.5 mg, thiamine; 2.3 mg riboflavin; 8.8 mg, pantothenic acid; 7 mg pyridoxine; 4 mg cobalamin; 0.016 mg niacin; 30 mg choline chloride; 650 mg biotin; 0.20 mg folic acid, 0.25 mg Mn; 40 mg Fe; 58 mg Zn; 40 mg Cu; 8 mg I, 0.60 mg Se.

3) Calculated values in DM basis.

**Table 2:** Effects of 0 (Control), 50 (EO-50), 100 (EO-100), and 200 mg/kg (EO-200) essential oil addition on apparent dry matter, organic matter and crude protein digestibility on 28th, 56th, and 84th day of the study, in DM %, (n = 20)

Days	Groups				SEM	p-value		
	
	Control	EO-50	EO-100	EO-200		ANOVA	Linear	Quadratic
Dry matter (d)								
28	69.74^b^	70.27^bB^	71.10^bB^	74.95^aB^	0.376	0.000	0.000	0.008
56	69.47^c^	72.06^bA^	73.08^bA^	75.11^aB^	0.317	0.000	0.000	0.535
84	69.93^c^	70.39^bcB^	71.84^bB^	75.97^aA^	0.374	0.000	0.000	0.001
SEM	0.253	0.295	0.418	0.259				
p-value	0.686	0.016	0.007	0.009				
Organic matter (d)								
28	73.93^c^	74.44^bcB^	75.99^b^	79.59^a^	0.396	0.000	0.000	0.016
56	74.41^c^	76.86^bA^	77.03^b^	79.16^a^	0.274	0.000	0.000	0.807
84	73.54^c^	75.06^cB^	76.94^b^	79.99^a^	0.383	0.000	0.000	0.170
SEM	0.264	0.400	0.348	0.174				
p-value	0.339	0.010	0.133	0.300				
Crude protein (d)								
28	50.08^c^	51.58^bcB^	53.58^bB^	56.22^aB^	0.474	0.000	0.000	0.483
56	51.29^d^	54.24^cA^	55.92^bA^	57.13^aAB^	0.321	0.000	0.000	0.041
84	51.06^c^	52.48^bcB^	54.70^bAB^	57.69^aA^	0.513	0.000	0.000	0.369
SEM	0.277	0.379	0.668	0.232				
p-value	0.127	0.019	0.029	0.000				

Represents the total number of birds used for nutrient digestibility parameters from each group, n = 20.

SEM, pooled standard error of means; ANOVA, analysis of variance.

a–d Means in a row with different superscripts differ significantly.

A,B Means in the same column with different superscripts differ significantly (p<0.05).

**Table 3:** Effects of 0 (Control), 50 (EO-50), 100 (EO-100), and 200 mg/kg (EO-200) essential oils addition on some serum biochemical parameters on 84th day of the study, (n = 10)

Items	Groups				SEM	p-value		
	
	Control	EO-50	EO-100	EO-200		ANOVA	Linear	Quadratic
Liver functions								
GGT (U/L)	26.1	26.6	26.7	26.9	0.48	0.950	0.580	0.882
AST (U/L)	147^b^	167^a^	166^a^	166^a^	2.80	0.022	0.019	0.061
ALT (U/L)	1.37	1.10	1.50	1.50	0.10	0.413	0.363	0.235
Renal and muscle function								
Creatinine (mg/dL)	0.39^b^	0.39^b^	0.41^b^	0.45^a^	0.01	0.001	0.000	0.061
Electrolytes								
Ca (mg/dL)	31.4^b^	33.3^a^	29.2^c^	31.2^b^	0.36	0.006	0.004	0.001
P (mg/dL)	5.31	5.79	5.99	5.42	0.12	0.168	0.095	0.641

SEM, standard errors of means; ANOVA, analysis of variance; GGT, gamma glutamyl transferase; AST, asparate aminotransferase; ALT, alanine aminotransferase.

a–c Means in a row with different superscripts differ significantly (p<0.05).

**Table 4:** Effects of 0 (Control), 50 (EO-50), 100 (EO-100), and 200 mg/kg (EO-200) essential oils addition on lymphocyte histochemistry and small intestinal morphology of laying hens on 84th day of the study (n = 6)

Item	Groups				SEM	p-value		
	
	Control	EO-50	EO-100	EO-200		ANOVA	Linear	Quadratic
Lymphocyte histochemistry								
ANAE positive PBL (%)	38.17^c^	58.17^a^	50.50^b^	56.00^ab^	1.97	0.000	0.000	0.007
ACP-ase positive PBL (%)	69.00	68.17	76.17	70.17	1.20	0.065	0.247	0.245
Intestinal morphology								
*Duodenum*								
VH (μm)	709.0^b^	698.9^b^	795.5^a^	813.8^a^	12.13	0.000	0.000	0.533
VW (μm)	86.51^a^	75.03^b^	77.09^b^	84.22^a^	1.25	0.002	0.650	0.000
CD (μm)	110.41^c^	93.69^d^	132.47^a^	119.22^b^	1.92	0.000	0.000	0.546
TTM (μm)	101.37^a^	77.39^c^	102.64^a^	88.96^b^	1.81	0.000	0.392	0.102
GCC (cell/100 μm)	6.28^a^	6.57^a^	6.28^a^	5.72^b^	0.07	0.000	0.001	0.001
*Jejunum*								
VH (μm)	600.2^a^	531.7^b^	583.1^a^	547.1^b^	6.31	0.000	0.042	0.169
VW (μm)	64.39^b^	66.47^b^	73.34^a^	70.25^ab^	1.08	0.015	0.010	0.218
CD (μm)	85.23^b^	77.04^c^	99.91^a^	69.87^d^	1.61	0.000	0.041	0.000
TTM (μm)	98.27^a^	90.67^a^	95.14^a^	82.47^b^	1.40	0.000	0.000	0.335
GCC (cell/100 μm)	6.30^c^	7.78^a^	8.10^a^	7.22^b^	0.09	0.000	0.000	0.000
*Ileum*								
VH (μm)	365.5	354.7	338.1	356.7	4.04	0.111	0.231	0.069
VW (μm)	51.42^d^	56.06^c^	68.35^a^	63.03^a^	1.00	0.000	0.000	0.003
CD (μm)	64.72^bc^	62.75^c^	70.03^ab^	71.20^a^	1.02	0.006	0.003	0.423
TTM (μm)	168.74^a^	154.58^a^	138.15^b^	163.27^a^	2.79	0.000	0.164	0.000
GCC (cell/100 μm)	8.08	8.45	8.62	8.27	0.08	0.090	0.304	0.022

SEM, standard errors of means; ANOVA, analysis of variance; ANAE, alpha-naphthyl acetate esterase; PBL, peripheral blood lymphocyte; ACP-ase, acid phosphatase; VH, villus height; VW, villus width; CD, crypt depth; TTM, thickness of tunica muscularis; GCC, goblet cell count.

a–d Means in a row with different superscripts differ significantly (p<0.05).
